# Plasma Membrane-Localized PtCOR8 Enhances Cold Tolerance in *Poncirus trifoliata* Through the ATCT Motif-Mediated Promoter Activation

**DOI:** 10.3390/plants15111743

**Published:** 2026-06-04

**Authors:** Na Li, Ben Zhang, Ling Gong, Cong He, Chunmiao Zhang, Xiang Liu, Suming Dai, Yingzi Zhang, Bing Wang, Guiyou Long, Dazhi Li

**Affiliations:** 1 College of Horticulture, Hunan Agricultural University, Changsha 410128, China; 1112lina@hunau.edu.cn (N.L.); zhangben711@163.com (B.Z.); 13873135667@163.com (L.G.); hecong2021@163.com (C.H.); 19929124010@163.com (C.Z.); lx2530365370@163.com (X.L.); dsm531@126.com (S.D.); zhangyingzi1005@163.com (Y.Z.); 2 Yuelushan Laboratory, Pomology Variety Innovation Center, Changsha 410128, China; 3 College of Plant Protections, Hunan Agricultural University, Changsha 410128, China

**Keywords:** *Poncirus trifoliata*, PtCOR8, cold tolerance, promoter regulation, ATCT motif

## Abstract

Cold stress is a critical abiotic factor that severely limits plant growth and agricultural productivity in subtropical regions. *Poncirus trifoliata* exhibits exceptional cold hardiness and is widely used as a rootstock in *Citrus*. However, the key genes and mechanisms conferring this resilience remain largely unexplored. Here, we characterized *PtCOR8*, a cold-induced gene isolated from *P. trifoliata*. Phylogenetic and subcellular localization analyses confirmed that *PtCOR8* encodes a plasma membrane-localized protein belonging to the WCOR413 family. Functional validation revealed that heterologous overexpression of *PtCOR8* in tomato significantly enhanced cold tolerance, concomitant with reduced malondialdehyde (MDA) content, elevated peroxidase (POD) activity, and upregulation of cold-responsive genes (e.g., *CIN8*). Notably, expression profiling of *COR8* in 16 citrus accessions under natural overwintering conditions indicated a strong positive correlation between its expression level and cold tolerance of different genotypes. Transgenic tomato plants with *PtCOR8* driven by its native promoter also presented enhanced cold tolerance, confirming that the native promoter is sufficient to drive functional expression under cold stress in the tomato system. Through promoter deletion and β-glucuronidase (GUS) staining experiments, the ATCT motif was further identified as a *cis*-acting element capable of mediating cold-induced promoter activity. Our findings uncover a dual-layered mechanism in which the PtCOR8 protein alleviates membrane lipid peroxidation and oxidative damage, while its transcription level is precisely modulated by a novel promoter regulatory mechanism, thereby improving freezing tolerance. This study provides important genetic insights and a valuable gene resource for cold-resistant citrus breeding.

## 1. Introduction

Low-temperature stress poses a severe threat to plant survival and productivity worldwide. Citrus, the most widely cultivated fruit crop and one of the most economically important horticultural commodities globally, is particularly vulnerable to chilling and freezing injuries [1]. China, as the world’s largest citrus producer, faces frequent cold events that threaten sustainable fruit production [2]. Temperature acts as a major abiotic factor restricting plant growth, development, and geographical distribution [3]. Citrus species are predominantly distributed between 30° S and 30° N, mainly in tropical and subtropical climates. In China, major citrus-producing areas lie south of the Yangtze River, whereas the southern Shaanxi region (31° N–33° N) marks the northern boundary of citrus cultivation, where the risk of low-temperature stress is considerably higher [4,5]. When ambient temperatures drop beyond the optimal range for citrus growth, normal physiological and biochemical processes are disrupted, leading to cellular injury and even plant death [6]. Periodic cold waves have repeatedly caused substantial yield losses. For instance, the severe snow disaster in 2008 inflicted extensive damage on major citrus-producing regions in China, including Hunan and Hubei Provinces [7]. Consequently, cold stress represents a major constraint on citrus distribution and high-quality cultivation, posing a persistent challenge to the global citrus industry [7]. Improving cold tolerance has thus become a primary breeding goal for citrus-producing countries worldwide.

Plants have developed complex adaptation strategies to survive under low-temperature stress [8,9,10]. Cold exposure triggers a series of physiological and molecular responses that reprogram cellular metabolism and gene expression [11,12]. A low-temperature environment could induce the expression of cold-responsive genes (*CORs*) in plants [13]. CORs generally refer to the proteins encoded by cold-responsive or cold-regulated genes, integrated into complex signal transduction frameworks that coordinate cellular responses to cold conditions [14]. Many *COR*s are activated via the *ICE*-*CBF*-*COR* signaling cascade, in which C repeat binding transcription factors (CBFs/DREBs) interact specifically with CRT/DRE *cis*-elements to trigger cold-induced transcription [15,16,17]. Multiple signaling molecules act synergistically through cross talk to modulate these pathways, including calcium (Ca^2+^) [18], reactive oxygen species (ROS) [19], and inositol phosphates [20].

Within this broader framework, the *COR413* gene family—unique to plants—was first identified in *Arabidopsis thaliana* as being involved in low-temperature stress response [21]. Members of the *COR413* family have since been cloned from multiple plant species, including Sea Island cotton [22] and salt-tolerant tamarisk [23]. Structurally, COR413 proteins include five conserved transmembrane domains, a potential glycosylphosphatidylinositol (GPI) anchor site, and a conserved phosphorylation motif, implying membrane-associated regulatory roles. COR413 plays a crucial role in the plant’s response to abiotic stress, particularly cold stress. For example, in *Chrysanthemum*, cold acclimation strongly upregulated *COR413*, and the magnitude of upregulation was significantly greater in the cold-tolerant *C. dichrum* than in the cold-sensitive *C. makinoi* [24].

In China, trifoliate orange (*Poncirus trifoliata*) is one of the most widely used rootstocks in citrus cultivation [25]. The use of *P. trifoliata* as a rootstock not only enhances the cold tolerance of grafted citrus varieties but also increases their resistance to certain diseases and pests [26]. Notably, this deciduous species can survive temperatures as low as −26 °C during winter, indicating its remarkable adaptability to cold climates [27]. In our previous work, we identified and cloned a cold-acclimation-related gene, *PtCOR8* (GenBank accession no. EU077497), from the leaves of *P. trifoliata* exposed to natural low-temperature conditions [28]. The present study aimed to elucidate the function of *PtCOR8* in cold tolerance and to identify the *cis*-acting elements within its promoter that mediate cold-induced gene expression. In this study, expression analysis revealed that *PtCOR8* transcripts accumulated to significantly higher levels in cold-tolerant citrus genotypes, whereas their expression remained relatively low in cold-sensitive varieties. Heterologous overexpression of *PtCOR8* in transgenic tomato plants (*Solanum lycopersicum*) resulted in a marked improvement of freezing tolerance, confirming its functional contribution to cold resistance. Further promoter analysis demonstrated that the *PtCOR8* promoter is activated by cold stress, and an ATCT motif within the promoter sequence serves as a *cis*-element responsible for mediating cold-induced transcriptional activation. Collectively, these findings identify *PtCOR8* as a promising candidate gene for improving cold tolerance in citrus through molecular breeding approaches, offering novel insights into the genetic and molecular basis of citrus cold adaptation.

## 2. Results

### 2.1. PtCOR8 Is Localized to the Plasma Membrane

**Figure 1:**
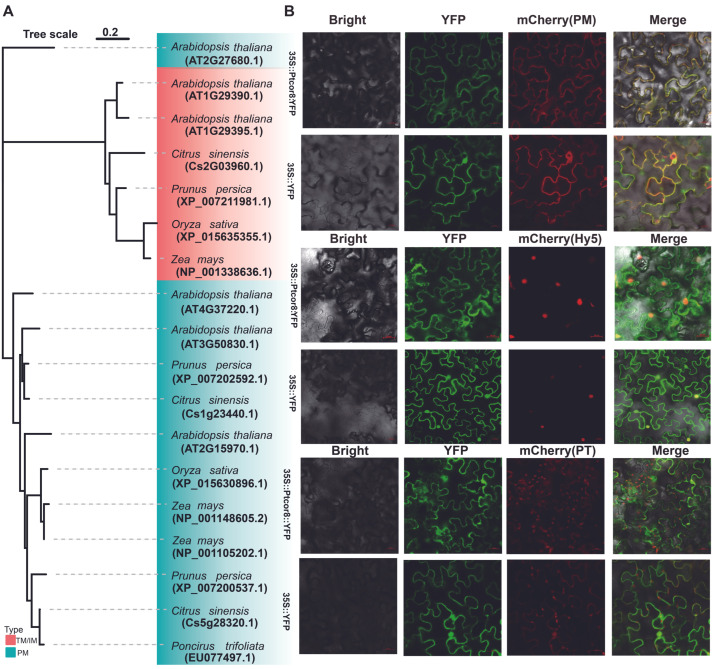
Phylogenetic analysis and subcellular localization of the PtCOR8. (**A**) Phylogenetic tree analysis of PtCOR8 and its homologous proteins in grape, *Arabidopsis*, apple, pear, and sweet orange. TM/IM indicates thylakoid membrane/inner membrane; PM indicates plasma membrane. (**B**) Subcellular localization of trifoliate orange PtCOR8. HY5 shows fluorescence localization of the nucleus marker; PM shows fluorescence localization of the plasma membrane marker; PT shows fluorescence localization of the plastid marker; GFP, green fluorescence; mCherry, red fluorescence; merge, merged image; scale bar, 10 μm.

PtCOR8 is classified as a member of the WCOR413 cold-induced protein family and encodes a 206-amino-acid protein derived from a 621 bp open reading frame (ORF). Phylogenetic analysis revealed that PtCOR8 clustered within the branch containing plasma membrane-associated COR413 proteins (Figure 1A, Supplementary Figure S2). To verify its cellular localization, we performed a subcellular localization assay using *N. benthamiana* leaf epidermal cells transiently expressing PtCOR8 fused to GFP under the control of the CaMV35S promoter. In parallel, fluorescent marker constructs for the plastid, plasma membrane, and nucleus were co-expressed to determine the exact localization pattern. Confocal microscopy analysis performed in *Nicotiana benthamiana* at standard growth temperature (22 °C) showed that the PtCOR8-GFP fusion protein co-localized with the plasma membrane marker, but not with the chloroplast or nuclear markers (Figure 1B). Calculation of colocalization coefficients gave a Pearson’s r of 0.904 ± 0.027, and Manders’ coefficients M1 and M2 were 0.999 and 0.993, respectively (Supplementary Table S3, Figure S7). In contrast, free GFP was uniformly distributed throughout the cytoplasm and nucleus of *N. benthamiana* cells. PtCOR8-GFP co-localized with the plasma membrane marker, and PtCOR8 was placed within the membrane-associated subgroup in the phylogenetic tree.

### 2.2. Heterologous Expression of PtCOR8 Enhances Cold Tolerance in Tomato

**Figure 2:**
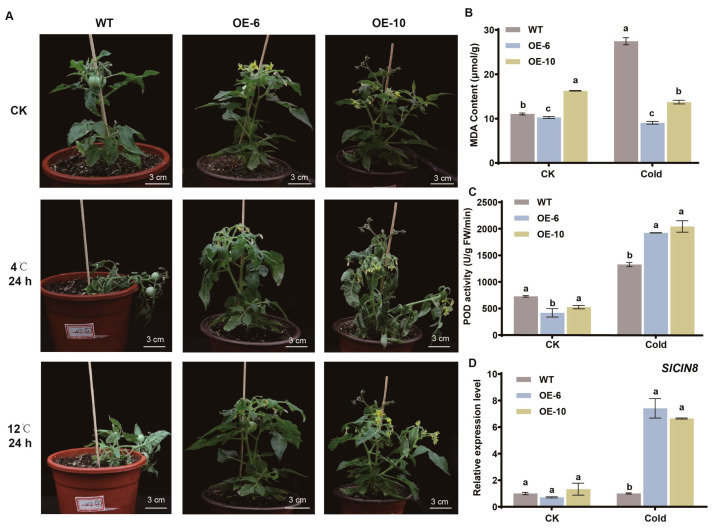
Low-temperature phenotype and physiological indices of *PtCOR8*-overexpressing tomato. (**A**) Phenotypic characteristics of *PtCOR8*-overexpressing tomato lines OE-6 and OE-10 under 4 °C low-temperature conditions. WT indicates wild-type tomato; OE-6 and OE-10 indicate *PtCOR8*-overexpressing tomato lines; CK represents the control sample prior to low-temperature treatment. Scale bar, 4 cm. (**B**) POD and MAD content and expression analysis of cold-related genes in *PtCOR8*-overexpressing tomato lines OE-6 and OE-10. WT indicates wild-type tomato; OE-6 and OE-10 indicate *PtCOR8*-overexpressing tomato lines; CK represents the control sample prior to low-temperature treatment; cold indicates a 24 h treatment at 4 °C. The data are expressed as mean ± standard deviation (3 biological replicates). Different lowercase letters indicate significant differences (*p* < 0.05) based on one-way ANOVA and Duncan’s new multiple range test (two-tailed). (**C**) Analysis of peroxidase (POD) in *PtCOR8*-overexpressing tomato lines OE-6 and OE-10. WT indicates wild-type tomato; OE-6 and OE-10 are *PtCOR8*-overexpressing lines; CK denotes untreated control before cold treatment, and Cold refers to 24 h treatment at 4 °C. Data are presented as mean ± standard deviation with three biological replicates. Significant differences (*p* < 0.05) are marked by different lowercase letters based on one-way ANOVA followed by two-tailed Duncan’s new multiple range test. (**D**) Analysis of cold-responsive gene expression in *PtCOR8*-overexpressing tomato lines OE-6 and OE-10. WT indicates wild-type tomato; OE-6 and OE-10 represent *PtCOR8*-overexpressing lines; CK stands for control before cold treatment, and Cold means treatment at 4 °C for 24 h. Data are shown as mean ± standard deviation (n = 3 biological replicates). Different lowercase letters denote significant differences at *p* < 0.05 according to one-way ANOVA and two-tailed Duncan’s new multiple range test.

To elucidate the functional role of *PtCOR8* in cold tolerance, we generated tomato (*S. lycopersicum*) lines overexpressing *PtCOR8* heterologously. A total of 10 independent transgenic lines were obtained. OE-6 and OE-10 showed higher expression levels based on quantitative and semi-quantitative PCR analyses (Figure S1) and were selected for further study. When subjected to cold treatment at 4 °C for 24 h, wild-type (WT) tomato plants exhibited darkened, dull leaves accompanied by severe wilting, whereas the *PtCOR8*-overexpressing plants showed only slight leaf darkening and milder wilting symptoms. After 12 h of recovery at 12 °C, transgenic plants resumed normal growth, while several leaves of the wild-type plants remained wilted (Figure 2A). Biochemical assays indicated that peroxidase (POD) activity in the transgenic lines OE-6 and OE-10 was 1.45- and 1.54-fold higher, respectively, than that in the wild type. In contrast, malondialdehyde (MDA) content exhibited an opposite trend, being 0.33- and 0.50-fold that of wild-type plants. Transgenic tomato plants overexpressing *PtCOR8* showed milder wilting, higher POD activity, and lower MDA content compared to wild-type (WT) tomato under cold stress (Figure 2B).

### 2.3. Expression of PtCOR8 Plays a Critical Role in Determining the Cold Tolerance of Citrus

**Figure 3:**
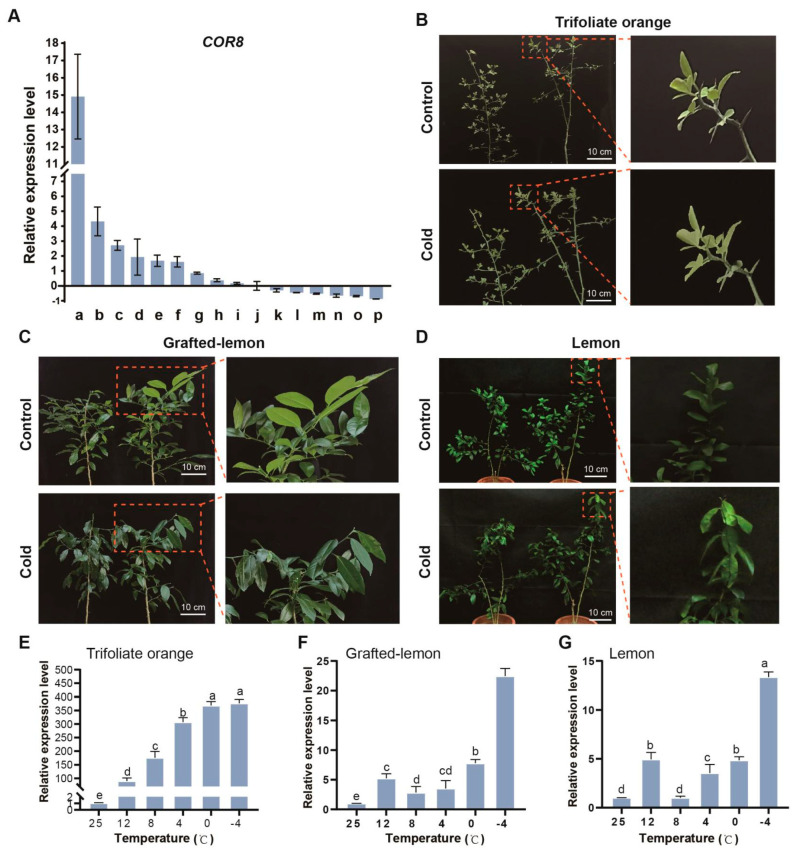
Relative expression characteristics of the *PtCOR8* gene. (**A**) Relative expression levels of the *COR8* in 16 citrus accessions with different cold tolerance during autumn–winter low-temperature acclimation, using ‘Newhall’ navel orange as the control. a–p represent the accessions trifoliate orange, ‘Cuimi’ kumquat, ‘Hunan’ sour orange, Citrumelo, Ichangensis, Citrange, ‘Shatian’ Pomelo, ‘Cocktail’ Grapefruit, Mangshanyeju, ‘Newhall’ navel orange, Citron ‘C-05’, ‘Lane Late’ navel orange, ‘Nanju’ Mandarin, ‘Juxianglong’ Bingtang sweet orange, ‘Eureka’ Lemon and ‘Femmninello S1’ Lemon. The data are expressed as mean ± standard deviation (3 biological replicates). In other citrus species, *COR8* is the orthologous gene of *PtCOR8*. (**B**–**D**) Low-temperature phenotypic results of trifoliate orange seedlings, grafted lemon seedlings, and lemon seedlings. Control represents the control sample prior to low-temperature treatment. Cold indicates treatment at 4 °C. (**E**–**G**) Expression levels of the *PtCOR8* in trifoliate orange seedlings, grafted lemon seedlings, and lemon seedlings after low-temperature treatment. The data are expressed as mean ± standard deviation (3 biological replicates. For each genotype, leaves collected from the same direction of two plants were pooled as one biological replicate, and three directional pools were generated). Different lowercase letters indicate significant differences (*p* < 0.05) based on one-way ANOVA and Duncan’s new multiple range test (two-tailed). Scale bar, 10 cm.

Alignment of PtCOR8 protein sequences from 16 citrus accessions showed they are highly conserved (Figure S2). Despite the highly conserved PtCOR8 protein sequences, *PtCOR8* transcript levels varied substantially between cold-tolerant and cold-sensitive varieties across the 16 citrus accessions. To further investigate the possible regulatory mechanism, we quantified *PtCOR8* transcript levels across the 16 citrus accessions. The expression of *PtCOR8* was markedly higher in cold-tolerant varieties than in sensitive ones, with the highest transcript abundance detected in *P. trifoliata*—approximately 14.91 times that of *C. sinensis* cv. Newhall (Figure 3A). *PtCOR8* transcript levels were positively correlated with cold tolerance among the citrus accessions. Furthermore, cold treatment was performed on *P. trifoliata*, lemon (*C*. *limon*), and lemon grafted onto *P. trifoliata* rootstock. The grafted lemons exhibited significantly enhanced cold tolerance compared with self-rooted lemons (Figure 3B). Correspondingly, *PtCOR8* expression was substantially upregulated in grafted lemon tissues under cold conditions (Figure 3C). Grafted lemons showed higher *PtCOR8* expression and enhanced cold tolerance compared to self-rooted lemons under cold conditions.

### 2.4. The PtCOR8 Promoter Enhances Cold Tolerance via Cold-Induced Expression

**Figure 4:**
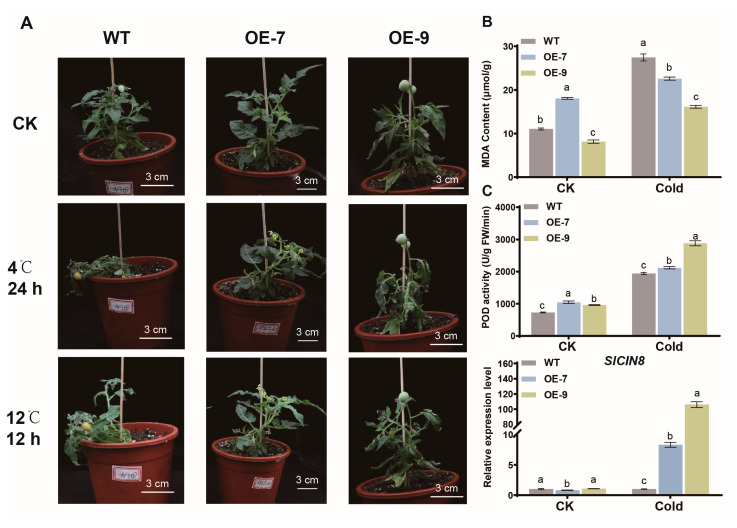
Low-temperature phenotype and physiological indices of tomato transformed with the native promoter p*PtCOR8*. (**A**) Phenotypic characteristics of tomato lines OE-7 and OE-9 transformed with the native promoter p*PtCOR8* under 4 °C low-temperature conditions. WT indicates wild-type tomato; OE-7 and OE-9 indicate tomato lines transformed with the native promoter p*PtCOR8*; CK represents the control sample prior to low-temperature treatment. (**B**) Measurements of POD and MDA content and expression analysis of cold-related genes in tomato lines OE-7 and OE-9 transformed with the native promoter p*PtCOR8*. WT indicates wild-type tomato; OE-7 and OE-9 indicate tomato lines transformed with the native promoter p*PtCOR8*; CK represents the control sample prior to low-temperature treatment. Cold indicates a 24 h treatment at 4 °C. The data are expressed as mean ± standard deviation (3 biological replicates). Different lowercase letters indicate significant differences (*p* < 0.05) based on one-way ANOVA and Duncan’s new multiple range test (two-tailed). (**C**) Determination of peroxidase (POD) contents in tomato lines OE-7 and OE-9 transformed with native promoter pPtCOR8. WT refers to wild-type tomato; OE-7 and OE-9 are transgenic lines carrying native pPtCOR8 promoter. CK is the control before low-temperature treatment, and Cold stands for 24 h treatment at 4 °C. Data are shown as mean ± standard deviation (3 biological replicates). Different lowercase letters indicate significant differences at *p* < 0.05 according to one-way ANOVA and two-tailed Duncan’s new multiple range test.

To investigate the regulatory role of the *PtCOR8* promoter in its cold-induced expression, a construct containing both the *PtCOR8* promoter region and its coding sequence (p*PtCOR8*-*PtCOR8*) was introduced into tomato (*S. lycopersicum*) for heterologous overexpression. Upon exposure to cold treatment at 4 °C for 24 h, the p*PtCOR8*-*PtCOR8* transgenic plants displayed significantly enhanced cold tolerance, with a phenotype consistent with that observed in 35S::*PtCOR8* overexpression lines. (Figure 4A,B). The *PtCOR8* promoter conferred cold-inducible expression and enhanced cold tolerance in transgenic tomato.

### 2.5. Mapping and Validation of a Core Cold-Responsive ATCT Motif in the PtCOR8 Promoter

**Figure 5:**
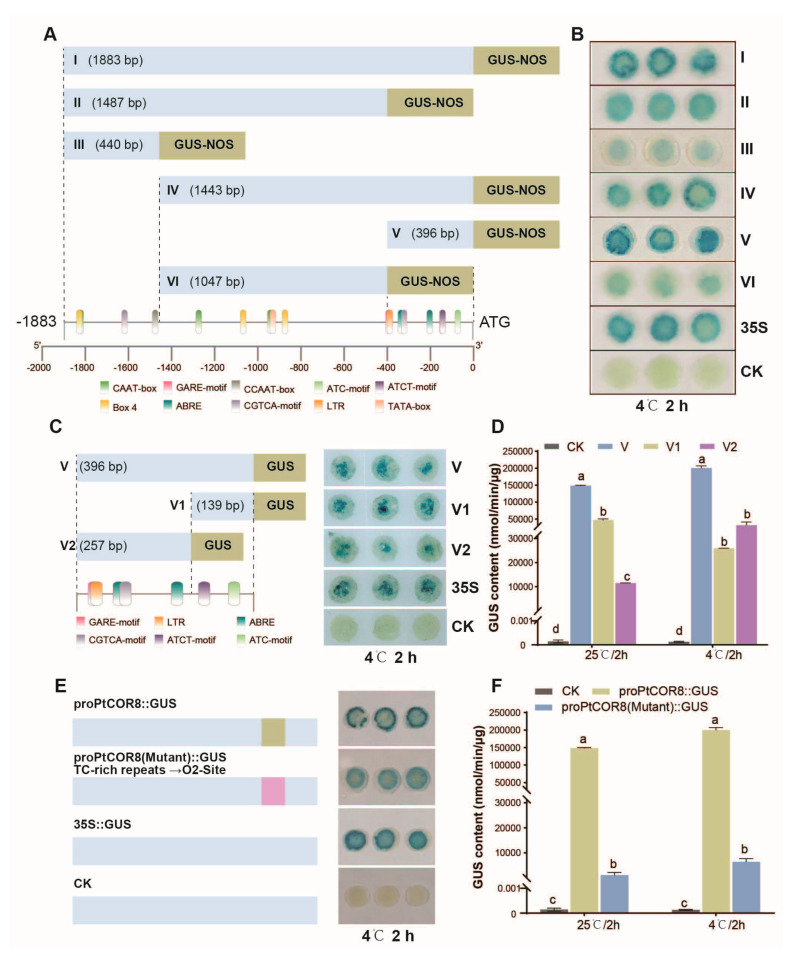
Cloning and activity analysis of *PtCOR8* promoter deletion fragments. (**A**) Schematic diagram of the vector backbone linking *PtCOR8* promoter deletion fragments to the GUS gene and the distribution of *PtCOR8* promoter elements. (**B**) Activity analysis of truncated *PtCOR8* promoters. I: p*PtCOR8* (−1883~−1 bp); II: p*PtCOR8* (−1883~−396 bp); III: p*PtCOR8* (−1883~−1443 bp); IV: p*PtCOR8* (−1443~−1 bp); V: p*PtCOR8* (−396~−1 bp); VI: p*PtCOR8* (−1443~−396 bp); 35S: positive control; CK: negative control. (**C**) GUS staining results of Pro*PtCOR8*-V deletion promoters under a 2 h treatment at 4 °C. V: Pro*PtCOR8*-V (−396~−1 bp); V1: Pro*PtCOR8*-V1 (−139~−1 bp); V2: Pro*PtCOR8*-V2 (−396~−139 bp); 35S: positive control; CK: negative control. (**D**) GUS reporter gene content of *PtCOR8* promoter deletion fragments. (**E**) Activity analysis of mutated *PtCOR8* promoters. (**F**) GUS reporter gene content of the truncated *PtCOR8* promoter fragment (−396~−1 bp) and the TC-rich repeats mutated fragment. The data are expressed as mean ± standard deviation (3 biological replicates). Different lowercase letters indicate significant differences (*p* < 0.05) based on one-way ANOVA and Duncan’s new multiple range test (two-tailed).

To identify the core region of the *PtCOR8* promoter responsible for cold responsiveness, we divided the promoter into six truncated fragments based on the predicted *cis*-acting elements and their positional distribution analyzed using PlantCARE (Figure 5A). The six fragments were named I (1883 bp), II (1487 bp), III (440 bp), IV (1443 bp), V (396 bp), and VI (1470 bp). To examine the effect of cold stress (4 °C) on promoter activity, each fragment was fused to a GUS reporter gene and transiently expressed in *N. benthamiana* leaves. After 48 h of exposure to 4 °C, GUS staining was performed to assess the promoter activity of different fragments. The negative control (CK) showed no blue staining, whereas the positive control (35S::GUS) exhibited a strong blue coloration, confirming the reliability of the assay. Among the truncated constructs, Pro*PtCOR8*-I (−1883~−1 bp), Pro*PtCOR8*-IV (−1443~−1 bp), and Pro*PtCOR8*-V (−396~−1 bp) displayed strong GUS activity, while Pro*PtCOR8*-II (−1883~−396 bp) and Pro*PtCOR8*-III (−1883~−1443 bp) exhibited weaker signals. These results suggest that the region −396~−1 bp likely represents the core cold-responsive segment of the *PtCOR8* promoter (Figure 5B).

To further investigate the key promoter region, we subdivided the −396 to −1 bp segment into two fragments: Pro*PtCOR8*-V1 (−139 to −1 bp) and Pro*PtCOR8*-V2 (−396 to −140 bp). These constructs were transiently expressed in *N. benthamiana* leaves via *Agrobacterium*-mediated infiltration, followed by 4 °C treatment for 2 h and GUS staining. The results showed that leaves transformed with Pro*PtCOR8*-V (−396~−1 bp) exhibited the deepest blue coloration (Figure 5C), followed by Pro*PtCOR8*-V1 (−139~−1 bp), whereas Pro*PtCOR8*-V2 (−396~−139 bp) showed a markedly lighter blue color. Further prediction revealed that the −139 to −1 bp promoter region contains two motifs, an ATCT motif and an ACT motif. The ATCT motif was initially reported as an important, conserved stress-responsive *cis*-element. Mutagenesis analysis revealed that Pro*PtCOR8*(ATCT motif mutant)::GUS resulted in noticeably weaker staining than Pro*PtCOR8*::GUS (Figure 5E). Pro*PtCOR8*-V and V1 exhibited strong GUS activity under cold conditions; mutation of the ATCT motif substantially reduced GUS staining.

## 3. Discussion

Cold stress imposes a severe limitation on citrus growth and productivity, particularly in subtropical regions where low-temperature episodes frequently occur during winter [29]. Plants have evolved complex molecular and physiological mechanisms to cope with cold-induced damage, among which COR proteins play vital roles in membrane stabilization and oxidative stress alleviation [30]. In this study, we identified *PtCOR8*, a member of the *WCOR413* family, as a *COR413* homolog that contributes significantly to cold tolerance in *Poncirus trifoliata* and its grafted citrus hybrids.

COR413 proteins are unique to plants and are categorized into three subclasses—COR413PM, COR413TM, and COR413IM—localized to the plasma membrane, thylakoid membrane, and chloroplast inner membrane, respectively [31]. The distinct subcellular localization of each subgroup implies functional divergence in stress adaptation. For example, overexpression of the *CpCOR413PM1* gene from wintersweet (*Chimonanthus praecox*) enhanced cold tolerance [32]. In this study, PtCOR8 was identified as a plasma membrane-localized COR413PM protein. The membrane localization of PtCOR8 suggests that it may directly participate in sensing and transducing cold signals.

Cold stress triggers ROS accumulation that can damage cellular components [33]; thus, ROS level and accumulation dynamics are useful indicators of stress sensitivity [34]. Plants counteract this oxidative burst by activating antioxidant enzymes such as SOD and POD and by extensive transcriptional reprogramming of stress-responsive genes to enhance stress tolerance [35,36,37]. For example, *PtrbHLH* of trifoliate orange plays a positive role in cold tolerance by modulating peroxidase (POD)-mediated H_2_O_2_ scavenging [38]. Functional characterization of *PtCOR8* through heterologous overexpression in tomato further confirmed its positive role in cold tolerance. Transgenic lines exhibited delayed wilting and significantly higher POD activity together with lower malondialdehyde (MDA) accumulation, demonstrating reduced oxidative damage under chilling conditions [39]. This finding is consistent with previous reports in other COR413 proteins that enhanced antioxidant enzyme activity under cold stresses, linking membrane protein function with redox regulation.

Regulation of cold-responsive (*COR*) gene expression plays a pivotal role in determining plant cold tolerance [40]. In cotton, *SikCOR413PM1* overexpression enhances cold stress tolerance in seedlings [41]. Similarly, two *Vitis amurensis VaCOR413* genes were significantly induced by low temperature, suggesting their involvement in cold-related metabolic pathways [42]. In citrus, comparative expression analyses revealed that transcriptional rather than structural variation underlies the differential cold responsiveness of *PtCOR8*, indicating that promoter regulation is the key determinant of its activation. *P. trifoliata*, renowned for its exceptional cold hardiness, exhibited the strongest *PtCOR8* induction after cold exposure. Intriguingly, lemon scions grafted onto *P. trifoliata* rootstocks showed markedly improved cold tolerance accompanied by higher *PtCOR8* transcript levels compared with self-rooted plants. These findings suggest that high expression of *PtCOR8* plays an important role in enhancing cold tolerance in citrus.

Promoters are key regulatory elements controlling gene expression and environmental responsiveness [43,44]. The *cis*-acting elements in the promoter region of a certain gene are essential factors for its expression [45]. Distinct *cis*-acting elements within the promoter are known to mediate responses to diverse upstream stresses. Many cold-responsive genes are transcriptionally activated by specific transcription factors that recognize cognate *cis*-regulatory elements upon cold exposure [46,47]. Our results showed that transgenic tomatoes overexpressing both the *PtCOR8* coding sequence and its native promoter region (p*PtCOR8*::*PtCOR8*) exhibited markedly improved cold tolerance compared with wild-type plants. We found that the promoter region of *PtCOR8* contained more than ten stress-responsive *cis*-acting elements, such as ABRE and CAAT-box. Reporter assays demonstrated that deletion or targeted mutation of ATCT motif in −139~−1 bp of *PtCOR8* promoter significantly reduced GUS staining intensity, confirming that the ATCT motif is crucial for full cold-responsive expression. The ATCT motif was initially reported as a conserved light-responsive cis-element, and its requirement for cold-induced *PtCOR8* expression suggests a potential interplay between light and cold signaling pathways.

## 4. Materials and Methods

### 4.1. Plant Materials

All citrus germplasm resources used in this study were grown at the National Citrus Improvement Center (Changsha), Hunan Agricultural University, located at longitude 113°09′ E and latitude 28°19′ N. Basic information on the experimental materials is provided in Supplementary Table S1.

For transient expression assays, *Nicotiana benthamiana* plants were employed, while tomato (*Solanum lycopersicum* cv. Micro Tom) was used for stable genetic transformation. Both species were cultivated in a growth chamber at Hunan Agricultural University under controlled environmental conditions: temperature 22 °C, light intensity 12,000 lx, photoperiod 16 h light/8 h dark, and relative humidity 60–70%.

Tomato (*Solanum lycopersicum* cv. Micro Tom) and *Nicotiana benthamiana* were treated at 4 °C to induce cold responses without causing freezing injury [48,49,50,51,52]. For citrus, a gradual decrease to −4 °C was applied to distinguish chilling stress from subzero freezing stress, taking advantage of the differential freezing tolerance between *Poncirus trifoliata* (tolerant) and *Citrus limon* (sensitive) [53,54].

### 4.2. Vectors and Strains

The pCAMBIA1300-35S-YFP vector was used to construct an overexpression vector and a vector driven by its native promoter (the PtCOR8 promoter), respectively. The overexpression vector was used for transient transformation of *N. benthamiana* and *S. lycopersicum*, whereas the native promoter-driven construct was used exclusively for stable transformation of Micro Tom tomato. For transient promoter activity assays, the promoter deletion fragments were ligated into the digested PCXSN-GUS vector via homologous recombination; this vector contained a β-glucuronidase (GUS) reporter gene and did not contain a leader peptide. All plasmids were maintained in our laboratory. The *Escherichia coli* strain DH10B and *Agrobacterium tumefaciens* strain GV3101, both prepared and maintained by our research group, were used for plasmid propagation and plant transformation, respectively.

### 4.3. cDNA Synthesis and RT-qPCR Analysis

Total RNA was extracted using the SteadyPure RNA Extraction Kit and subsequently reverse-transcribed into cDNA with the Evo M-MLV RT Master Mix (Hunan Accurate Bioengineering Co., Ltd., Changsha, China) according to the manufacturer’s instructions. Quantitative real time PCR (RT-qPCR) was performed on a Bio-Rad Laboratories, Inc., Hercules, CA, USA/C1000 Touch Thermal Cycler. using a total reaction volume of 10 μL, which contained 5 μL of 2× qPCR Master Mix, 0.2 μL of each primer (10 μM), 1 μL of 10× diluted cDNA, and 3.6 μL of nuclease-free water. The amplification program consisted of an initial denaturation at 95 °C for 2 min, followed by 36 cycles of 95 °C for 5 s and 60 °C for 30 s, and a final extension at 95 °C for 15 s. A melting curve analysis was performed at the end of each run to verify the specificity of amplification. The actin gene was used as an internal reference, and the relative expression level of each target gene was calculated using the 2^−ΔΔCT^ method. All reactions were conducted in three independent biological replicates to ensure reproducibility.

### 4.4. Low-Temperature Expression Characteristics of the COR8 Gene

To investigate the expression pattern of *COR8* under natural cold conditions (see Supplementary Figure S4), leaf samples of 16 citrus accessions—trifoliate orange (*Poncirus trifoliata* (L.) Raf.), Citrange (*Citrus* × *Poncirus* hybrid), Citrumelo (*Citrus* × *Poncirus* × *Citrus maxima* (Burm.) Merr. hybrid), ‘Cuimi’ kumquat (*Fortunella* × *Citrus* ‘Cuimi’), ‘Nanju’ Mandarin (*Citrus reticulata* Blanco ‘Nanju’), ‘Juxianglong’ Bingtang sweet orange (*Citrus sinensis* (L.) Osbeck ‘Juxianglong’), ‘Lane Late’ navel orange (*Citrus sinensis* (L.) Osbeck ‘Lane Late’), ‘Newhall’ navel orange (*Citrus sinensis* (L.) Osbeck ‘Newhall’), ‘Shatian’ Pomelo (*Citrus maxima* (Burm.) Merr. ‘Shatian’), ‘Cocktail’ Grapefruit (*Citrus paradisi* Macfad. ‘Cocktail’), ‘Eureka’ Lemon (*Citrus limon* (L.) Burm. f. ‘Eureka’), ‘Femmninello S1’ Lemon (*Citrus limon* (L.) Burm. f. ‘Femmninello S1’), Citron ‘C-05’ (*Citrus medica* L. ‘C-05’), ‘Hunan’ sour orange (*Citrus aurantium* L. ‘Hunan’), Ichang papeda (*Citrus ichangensis* Swingle), Mangshanyeju (*Citrus reticulata* ‘Mangshan’) (see Supplementary Table S1)—were collected from current-year spring shoots between September 2019 and January 2020 at 15 day intervals, resulting in nine sampling time points. All collected leaves were immediately frozen in liquid nitrogen and stored at −80 °C until RNA extraction. The relative transcript levels of *COR8* were quantified by RT-qPCR using specific primers listed in Supplementary Table S2.

For controlled low-temperature treatments, two plants each of the cold-tolerant germplasm *P. trifoliata*, the cold-sensitive seedling lemon (*C. limon*), and grafted lemon (six plants in total) were grown in an artificial climate chamber (model LEDR 400, Ningbo Yanghui Instruments, Ningbo, China) under 16 h light/8 h dark, 85% relative humidity, and 26 °C for 7 days of pre-conditioning. Subsequently, the temperature was decreased from 26 °C to 12 °C at a rate of 1 °C h^−1^ and then maintained at 12 °C, 8 °C, 4 °C, 0 °C, and −4 °C for 24 h each.

Leaf samples were collected before treatment (25 °C) and after each temperature gradient stage. For each plant, nine mature and healthy leaves were sampled from the third to fifth nodes of shoots in three directions (three leaves per direction). Leaves collected from the same direction of the two plants within each genotype were pooled as one biological replicate, yielding three biological replicates per genotype. The expression level of *PtCOR8* was determined by RT-qPCR using the primer pairs shown in Supplementary Table S2, with the pre-treatment sample as the control.

### 4.5. Subcellular Localization of PtCOR8

The full-length coding sequence of *PtCOR8* without the stop codon was cloned into the pCAMBIA1300-35S-YFP vector to generate the PtCOR8-YFP fusion construct. The resulting plasmid was introduced into *N. benthamiana* leaves via the *Agrobacterium tumefaciens* strain GV3101 using the agro-infiltration method. After 48 h of incubation, YFP fluorescence was observed using a Zeiss LSM 900 confocal laser scanning microscope (Carl Zeiss AG, Oberkochen, Germany). Excitation was performed at 488 nm, and fluorescence emission was collected between 500 nm and 550 nm. Colocalization analysis was performed on four independent fields of view using ImageJ (Version 1.54t 16 May 2026) JACoPv2.0 (Costes’ automatic threshold, 1000 randomizations) to obtain Pearson’s r and Manders’ M1/M2 coefficients.

### 4.6. Genetic Transformation of Tomato

The full-length coding sequence of *PtCOR8* was amplified using the primers listed in Supplementary Table S2 and inserted into the pCAMBIA1300 vector under the control of the CaMV 35S promoter to generate the overexpression construct (pCAMBIA1300::35S::PtCOR8). To obtain a native promoter-driven construct, the endogenous PtCOR8 promoter sequence replaced the 35S promoter in pCAMBIA1300, after which the PtCOR8 coding sequence was inserted downstream of the promoter, yielding pCAMBIA1300::pPtCOR8::PtCOR8.

Tomato (*Solanum lycopersicum* cv. Micro Tom) plants were transformed via *A. tumefaciens* mediated transformation according to [55]. The transgenic and wild-type (WT) plants were grown in a greenhouse, with the temperature maintained at 25 °C and a 16 h light/8 h dark photoperiod until phenotypically stable.

After the plants reached a stable growth stage, total RNA was isolated from leaves, and the expression of *PtCOR8* was analyzed by RT-qPCR using the primer pairs listed in Supplementary Table S2. Based on the RT-qPCR results, three P1300::35S::PtCOR8 transgenic lines that displayed significant transcriptional responses to cold stress (OE 4, OE 6, and OE 10), together with WT plants, were selected for semi-quantitative PCR (semi-qPCR) analysis. For each tomato line, cDNA synthesized from total RNA served as the template, and the same primers used for RT-qPCR were adopted in semi-qPCR assays.

### 4.7. Determination of Physiological and Biochemical Parameters

The malondialdehyde (MDA) content was measured based on the reaction between MDA and thiobarbituric acid (TBA) under high temperature and acidic conditions. The peroxidase (POD) activity was assayed using the guaiacol oxidation method as outlined by Guo et al. [56].

### 4.8. Promoter Truncation and Site-Directed Mutation of PtCOR8

According to our previous research [57], the *PtCOR8* promoter sequence was analyzed based on the distribution of its characteristic CCAAT box and ATCT motif elements. Using the primers listed in Supplementary Table S2, an 1883 bp upstream fragment from the translation start site of *PtCOR8* was amplified and systematically truncated to generate six promoter fragments of 1883 bp, 1487 bp, 1443 bp, 1047 bp, 440 bp, and 396 bp. These fragments were designated ProPtCOR8 I to VI, respectively.

For mutation analysis, the full-length promoter fragment ProPtCOR8 I (−1883 to −1 bp) was used as a template. Site-directed mutagenesis by overlapping extension PCR was performed following the method described by Heckman and Pease [58]. Using the primers listed in Supplementary Table S2, the ATCT motif within ProPtCOR8 I was mutated. Following three rounds of PCR amplification, the expected mutated promoter fragment was successfully obtained.

### 4.9. GUS Staining and Quantitative Analysis of GUS Reporter Gene Activity

Homologous recombination technology was used to ligate the truncated and mutated promoter fragments with the pCXSN-GUS reporter vector. The resulting recombinant plasmids were introduced into *N. benthamiana* leaves via *A. tumefaciens* strain GV3101-mediated transient transformation. After 48 h of infiltration, the inoculated tobacco plants were transferred to a 4 °C growth chamber for cold treatment. Leaf disks with a diameter of 1 cm were collected at 0, 2, 6, and 12 h of cold exposure for histochemical GUS staining, following the procedure described by Xie et al. [59]. The GUS enzymatic activity was quantified using the GUS Gene Quantitative Detection Kit (Beijing Coolaber Technology Co., Ltd., Beijing, China) in conjunction with the Bradford Protein Assay Kit, following the manufacturer’s instructions.

### 4.10. Statistical Analysis

SPSS 24.0 and GraphPad Prism 8.0.2 software were used for all statistical analyses. Data were analyzed by one-way ANOVA followed by Duncan’s new multiple range test (two-tailed) for post hoc multiple comparisons. Different lowercase letters indicate significant differences at *p* < 0.05.

## 5. Conclusions

**Figure 6:**
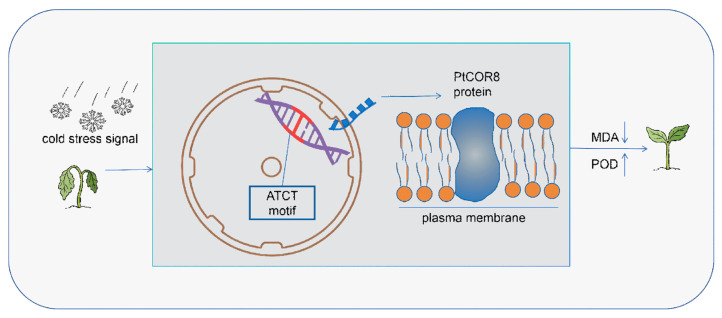
A dual-layer regulatory mechanism of PtCOR8 in enhancing freezing tolerance.

In summary, *PtCOR8* enhances cold tolerance in citrus primarily by mitigating oxidative damage under low-temperature stress. The expression level of *PtCOR8* is a key determinant of improved frost hardiness among citrus species. Mechanistically (See Figure 6), the *PtCOR8* promoter senses cold signals via the ATCT motif within its −139 to −1 bp region, enabling rapid transcriptional activation upon exposure to low temperatures. The identification of this functional ATCT motif not only clarifies the molecular basis of cold-induced gene expression but also provides a potential strategy for targeted breeding and promoter engineering to improve cold tolerance in economically important citrus cultivars. Future studies are needed to identify the transcription factors that directly bind this motif under cold stress and to determine whether light signaling components contribute to its cold-dependent activation.

## Data Availability

Data are contained within the article and Supplementary Materials.

## Supplementary Materials

The following supporting information can be downloaded at: https://www.mdpi.com/article/10.3390/plants15111743/s1, Figure S1: Analysis of *PtCOR8* gene expression level and semi-quantitative analysis in overexpressing tomato; Figure S2: Conserved domain analysis of *COR* gene in 16 citrus accessions; Figure S3: Amino acid sequence alignment of COR genes from 16 citrus accessions revealed that COR8 in other citrus species is the orthologous gene of PtCOR8; Figure S4: Natural overwintering temperature parameters from September 2019 to January 2020; Figure S5: pCAMBIA1300-35S-YFP vector map used for tomato transgenesis; Figure S6: PCXSN-GUS map used for promoter transient activity validation; Figure S7: Dot plot of colocalization coefficients. Table S1: 16 Citrus accessions resources with different cold tolerance; Table S2: Primers used in the experiment; Table S3: Co-localization coefficients of PM vs. PtCOR8-GFP.
