# Supplementary material for: Plasma Membrane-Localized PtCOR8 Enhances Cold Tolerance in Poncirus trifoliata Through the ATCT Motif-Mediated Promoter Activation
Source: Plants (Basel). 2026 Jun 4;15(11):1743. doi: 10.3390/plants15111743 (PMC13258929; doi:10.3390/plants15111743)
Supplement: Supplementary file 1 [file plants-15-01743-s001.zip › plants-4332740-supplementary.pdf]

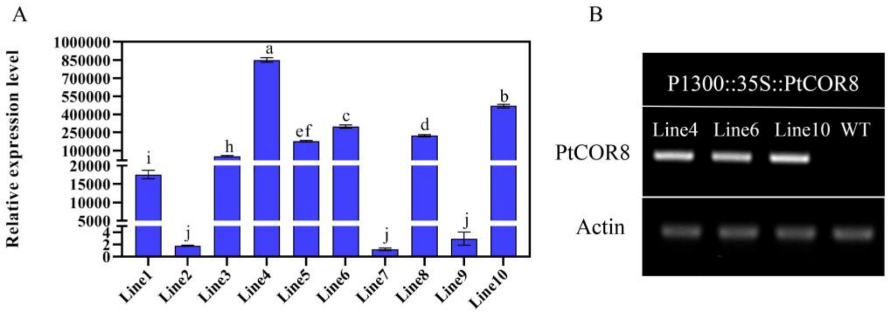

Supplementary Figure S1 Analysis of *PtCOR8* gene expression level and semi-quantitative analysis in overexpressing tomato.

A) Analysis of *PtCOR8* gene expression level in overexpressing tomato plants. Line1–Line10 indicate *PtCOR8*-overexpressing tomato lines; error bars represent standard errors among three biological replicates; different lowercase letters indicate significant differences ($p < 0.05$). B) Semi-quantitative analysis of tomato transformed with *P1300-35S-PtCOR8*. WT indicates wild-type tomato; Line4, Line6, and Line10 indicate *PtCOR8*-overexpressing tomato lines.

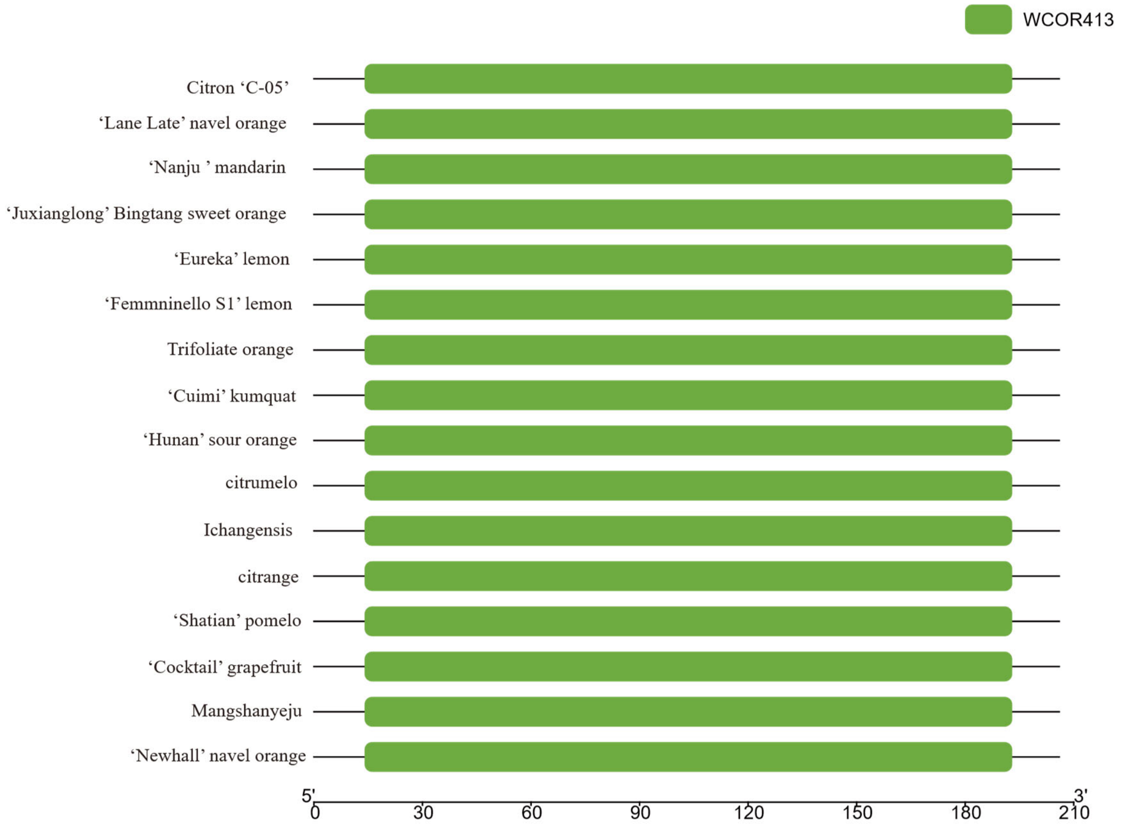

Supplementary Figure S2 Conserved domain analysis of *COR* gene in 16 citrus accessions

Supplementary Table S1 16 Citrus accessions resources with different cold tolerance

Code	Selected material	Scientific Name	Seedlings types	Age
1	Trifoliate orange	*Poncirus trifoliata*	seedling	6 years
2	Citrange	*Citrus* × *Poncirus*	seedling	9 years
3	Citrumelo	*Citrus* × *Poncirus* × *Citrus maxima*	seedling	9 years
4	Cuimi kumquat	*Fortunella* × *Citrus* ‘Cuimi’	Grafted seedlings (Poncirus trifoliata as rootstock)	5 years
5	Nanju mandarin	*Citrus reticulata* ‘Nanju’	Grafted seedlings (Poncirus trifoliata as rootstock)	12 years
6	Juxianglong Bingtang Sweet Orange	*Citrus sinensis* ‘Juxianglong’	Grafted seedlings (Poncirus trifoliata as rootstock)	12 years
7	Lane Late Navel Orange	*Citrus sinensis* ‘Lane Late’	Grafted seedlings (Poncirus trifoliata as rootstock)	4 years
8	Newhall Navel Orange	*Citrus sinensis* ‘Newhall’	Grafted seedlings (Poncirus trifoliata as rootstock)	4 years
9	Shatian pomelo	*Citrus maxima* ‘Shatian’	Grafted seedlings (Poncirus trifoliata as rootstock)	4 years
10	Cocktail Grapefruit	*Citrus* × *paradisi* ‘Cocktai’	Grafted seedlings (Poncirus trifoliata as rootstock)	4 years
11	Eureka lemon	*Citrus limon* ‘Eureka’	Grafted seedlings (Poncirus trifoliata as rootstock)	7 years
12	Femmninello S1lemon	*Citrus limon* ‘Femmninello S1’	Grafted seedlings (Poncirus trifoliata as rootstock)	7 years
13	Citron C-05	*Citrus medica* ‘C-05’	Grafted seedlings (Poncirus trifoliata as rootstock)	7 years
14	Hunan Sour orange.	*Citrus aurantium* ‘Hunan’	Grafted seedlings (Poncirus trifoliata as rootstock)	20 years
15	Ichangensis	*Citrus ichangensis*	Grafted seedlings (Poncirus trifoliata as rootstock)	15 years
16	Mangshanensis	*Citrus reticulata* ‘Mangshan’	Grafted seedlings (Poncirus trifoliata as rootstock)	15 years

Supplementary Table S2 Primers used in the experiment

Primer Name	Primer Sequences(5’-3’)	Use
PtCOR8-F	gagaacacgggggacgagctcATGATGGGTAAGAAGAGT	Amplify the *PtCOR8* gene sequence
PtCOR8-R	gcccttgctcaccatggtaccTAGGATGTAAAGCACCAG	
P1300-R	GCCGGACACGCTGAACTTGT	Positive identification of pCAMBIA1300-35S-YFP vector
p*PtCOR8*-F	tatgaccatgattacgaattcATGCCCCGATCTGTAGGGAA	Amplify the *PtCOR8* promoter sequence
p*PtCOR8*-R	cttcttacccatcatgagctcACAACTCAAGAAACAGCAAGAAGC	
Hyg-F	CCATACAAGCCAACCACG	Vector primers for identifying genetically modified tomatoes
Hyg-R	CTATTGCATCTCCCGCCG	
SlActin-F	AAGTGCGAGTGTCCTGTCTG	Reference genes for detecting the expression level of genetically modified tomatoes
SlActin-R	TACCGTGCATTCATAGCCCC	
q-*SlCIN8*-F	TCAACAACCGTCTCAGTGC	Detect the expression level of the *SlCIN8* gene in genetically modified tomatoes
q-*SlCIN8*-R	TCCAATCCACAAGCCAAG	
q-*PtCOR8*-F	TGGTTGCTCCCAGTTT	Detect the expression level of the *PtCOR8* gene in genetically modified tomatoes
q-*PtCOR8*-R	GCCCAGGCAGGATAGA	
I-F	aggatccccaatactATGCCCCGATCTGTAGGGAA	Full-length promoter cloning
I-R	agtattggggatccaACAACTCAAGAAACAGCAAGAAGC	
II-F	aggatccccaatactATGCCCCGATCTGTAGGGAA	Cloning of Missing Fragment II
II-R	agtattggggatccaGACACCTGCCATTGCCATCA	
III-F	aggatccccaatactATGCCCCGATCTGTAGGGAA	Cloning of Missing Fragment III
III-R	agtattggggatccaGGTGTGTGGGCTGATGTTAG	
IV-F	aggatccccaatactCTAACATCAGCCCACACACC	Cloning of the missing fragment IV
IV-R	agtattggggatccaACAACTCAAGAAACAGCAAGAAGC	
V-F	aggatccccaatactTGATGGCAATGGCAGGTGTC	Cloning of Missing Fragment V
V-R	agtattggggatccaACAACTCAAGAAACAGCAAGAAGC	
VI-F	aggatccccaatactCTAACATCAGCCCACACACC	Cloning of Missing Fragment VI
VI-R	agtattggggatccaGACACCTGCCATTGCCATCA	
V1-F	aggatccccaatactTTTAGATTAGATTACAATCACAGC	Cloning of the missing fragment

	ACAAACCG	V1
V2-F	aggatccccaatactTGATGGCAATGGCAGGTGTC	Cloning of the missing fragment
V2-R	tggatccccaatactTCTAATTGCCACGTGGACG	V2
I-F	aggatccccaatactATGCCCCGATCTGTAGGGAA	Cloning of point mutant
T1-R	CCATGTCATCATATCAACATCTAATTGCCACGTGG	ATCT-motif 1
T2-F	GATGACATGGTTACAATCACAGCACAAACCGAC	Cloning of point mutation ATCT-motif fragment 2
I-R	agtattggggatccaACAACTCAAGAAACAGCAAGAAGC	Cloning of deletion fragment V1 and point mutation ATCT-motif fragment 1

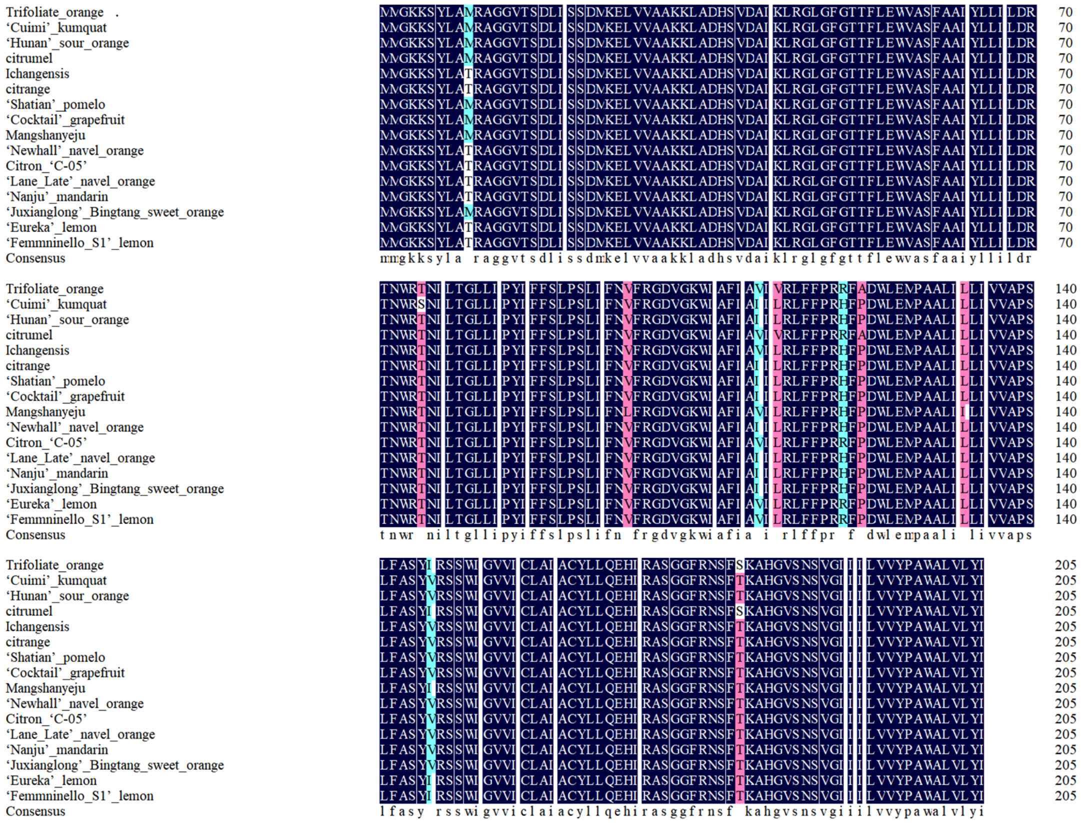

Supplementar Figure S3 Amino acid sequence alignment of COR genes from 16 citrus accessions revealed that COR8 in other citrus species is the orthologous gene of PtCOR8.

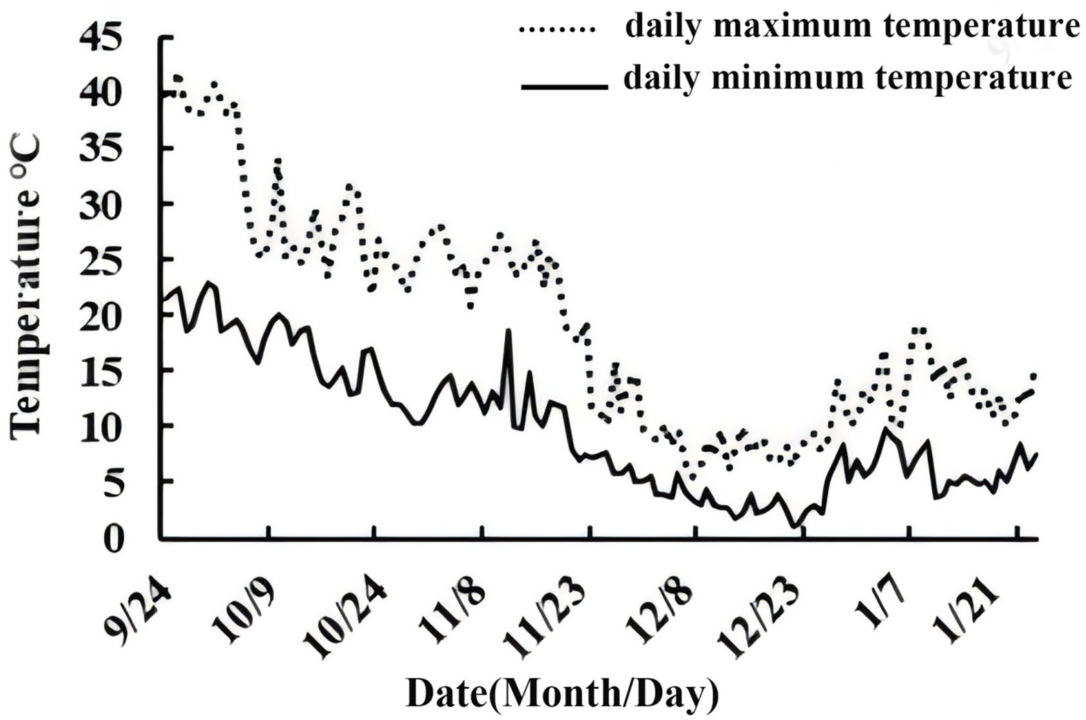

Supplementar Figure S4 Natural overwintering temperature parameters from September 2019 to January 2020

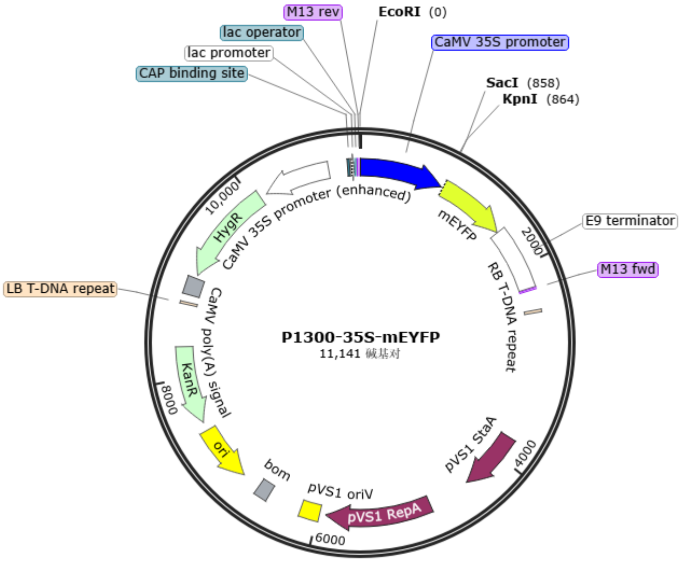

Supplementar Figure S5 pCAMBIA1300-35S-YFP vector map used for tomato transgenesis

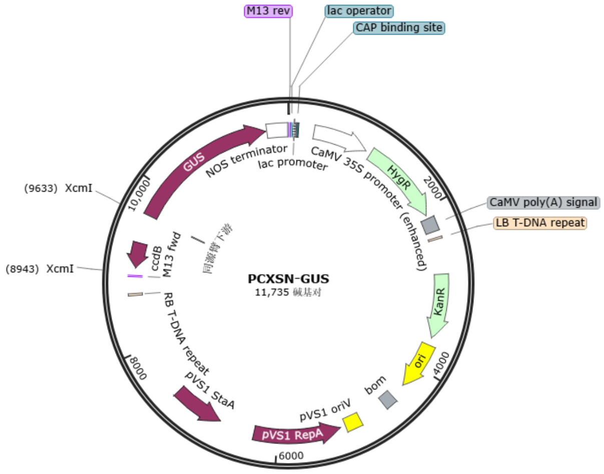

Supplementary Figure S6 PCXSN-GUS map used for promoter transient activity validation.

Supplementary Table S3. Co-localization coefficients of PM vs. PtCOR8-GFP

Field	Pearson’s r	M1	M2
1（used in manusctipt）	0.945	0.999	0.999
2	0.885	0.999	0.995
3	0.898	0.999	0.995
4	0.888	0.999	0.981
Mean ± SD	0.904±0.027	0.999±0.000	0.993±0.008

Note：M1 = fraction of PM overlapping PtCOR8-GFP, M2 = fraction of PtCOR8-GFP overlapping PM. Costes randomization P = 100% for all fields.

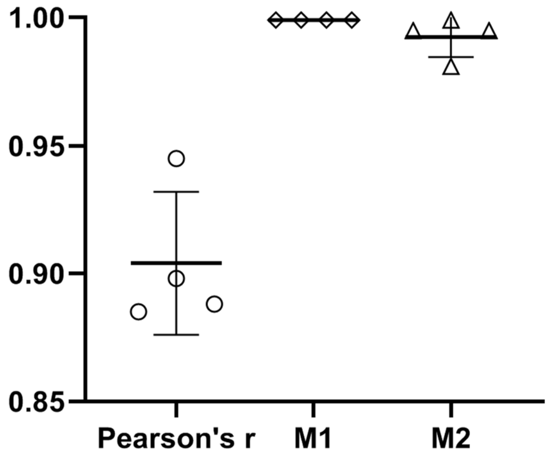

Figure S7. Dot plot of colocalization coefficients

Note： Dot plot showing Pearson’s r, M1 and M2 for PM vs. PtCOR8-GFP across four fields of view. Each dot represents one field. Colocalization analysis was performed using JACoP (Costes automatic threshold + 1000 randomizations). All Costes P-values = 100%.
